# Modeling Studies of the Mechanism of Context-Dependent Bidirectional Movements of Kinesin-14 Motors

**DOI:** 10.3390/molecules29081792

**Published:** 2024-04-15

**Authors:** Ping Xie

**Affiliations:** Key Laboratory of Soft Matter Physics, Institute of Physics, Chinese Academy of Sciences, Beijing 100190, China; pxie@aphy.iphy.ac.cn

**Keywords:** molecular motor, kinesin, movement direction, chemo–mechanical coupling mechanism

## Abstract

Kinesin-14s, a subfamily of the large superfamily of kinesin motor proteins, function mainly in spindle assembly and maintenance during mitosis and meiosis. KlpA from *Aspergillus nidulans* and GiKIN14a from *Giardia intestinalis* are two types of kinesin-14s. Available experimental results puzzlingly showed that while KlpA moves preferentially toward the minus end in microtubule-gliding setups and inside parallel microtubule overlaps, it moves preferentially toward the plus end on single microtubules. More puzzlingly, the insertion of an extra polypeptide linker in the central region of the neck stalk switches the motility direction of KlpA on single microtubules to the minus end. Prior experimental results showed that GiKIN14a moves preferentially toward the minus end on single microtubules in either tailless or full-length forms. The tail not only greatly enhances the processivity but also accelerates the ATPase rate and velocity of GiKIN14a. The insertion of an extra polypeptide linker in the central region of the neck stalk reduces the ATPase rate of GiKIN14a. However, the underlying mechanism of these puzzling dynamical features for KlpA and GiKIN14a is unclear. Here, to understand this mechanism, the dynamics of KlpA and GiKIN14a were studied theoretically on the basis of the proposed model, incorporating potential changes between the kinesin head and microtubule, as well as the potential between the tail and microtubule. The theoretical results quantitatively explain the available experimental results and provide predicted results. It was found that the elasticity of the neck stalk determines the directionality of KlpA on single microtubules and affects the ATPase rate and velocity of GiKIN14a on single microtubules.

## 1. Introduction

Kinesin-14s constitute a subfamily of the large superfamily of motor proteins that can interact with microtubules (MTs) [1,2,3,4]. Kinesin-14s function mainly in spindle assembly and maintenance during mitosis and meiosis [5,6,7,8,9,10]. Most of them, such as *Drosophila* Ncd, human HSET, KlpA from *Aspergillus nidulans*, GiKIN14a from *Giardia intestinalis*, etc., are homodimers, containing two identical C-terminal motor domains (also called heads), a neck extending from the two heads and a tail domain at the N-terminus [11,12,13,14]. This paper focuses mainly on KlpA and GiKIN14a.

Qiu and his colleagues [15,16] experimentally studied the dynamics of KlpA (see Table 1). They found that the truncated construct of KlpA, lacking an N-terminal tail domain, behaves like other non-processive kinesin-14s, with the motor mostly interacting with a single MT in a diffusive manner with no apparent directional preference [15]. In MT gliding by KlpA, with the N-terminus of its neck stalk or its tail domain anchored on a fixed surface, the motor exhibits minus-end-directed motility [15], also other kinesin-14s. Inside the parallel MT overlap, KlpA moves preferentially toward and gradually accumulates at the minus ends [15]. Intriguingly, on a single MT, KlpA exhibits plus-end-directed processive motility [15]. It was found that KlpA contains an intrinsically flexible central region in its neck stalk [16], indicating that its tail and head can simultaneously interact with the same MT. These results indicate that the tail of KlpA is a directionality-switching factor: to achieve plus-end-directed processive motility, the tail and head are required to bind to the same MT, and to achieve minus-end-directed motility, the tail is required to detach from the MT to which the head binds. Further experiments showed that the insertion of an extra polypeptide linker (3 × GS) in the central region of the neck stalk of KlpA switches the motility direction toward the minus end when the tail and head can bind to the same MT [16]. This indicates that the neck stalk is also a directionality-switching factor. Therefore, a critical issue is what the underlying mechanism is behind the phenomenon that the two factors control the motility direction of KlpA.

Tseng et al. [17] experimentally studied the dynamics of a single GiKIN14a moving on a single MT. Interestingly, they found that the tailless GiKIN14a is a minimally processive motor that can move toward the minus end, like human HSET [18]. The single full-length GiKIN14a can also move toward the minus end, with a processivity much longer than the tailless GiKIN14a. It was found that GiKIN14a also contains an intrinsically flexible central region in its neck stalk [17], like KlpA. This indicates that the additional interaction of the tail with MTs can enhance significantly the processivity of GiKIN14a. More intriguingly, Tseng et al. [17] found that full-length GiKIN14a has a larger ATPase rate and velocity than the tailless GiKIN14a. Moreover, the insertion of an extra polypeptide linker (3 × GS) in the central region of the neck stalk of GiKIN14a reduces the ATPase rate [17].

However, the above-mentioned experimental results have not been explained quantitatively up to now. How does KlpA exhibit its canonical minus-end-directed motility in MT gliding whereas it exhibits non-canonical plus-end-directed motility on a single MT? How does KlpA move toward the plus end on the single MT, where the tail domain and head can interact with the same MT, whereas move toward the minus ends inside the parallel MT overlap, where the tail domain and head can interact with different MTs? How does the insertion of an extra polypeptide linker (3 × GS) in the central region of the neck stalk of KlpA switch its motility direction to the minus end when its tail domain and head can interact with the same MT? How does the tail domain accelerate the ATPase rate and velocity of GiKIN14a on a single MT? How does the insertion of an extra polypeptide linker (3 × GS) in the central region of the neck stalk of GiKIN14a reduce its ATPase rate on a single MT? How does the insertion of the extra 3 × GS in the central region of the neck stalk of GiKIN14a affect its velocity on a single MT? In this paper, we address the above-mentioned unclear issues. For this purpose, we theoretically studied the dynamics of kinesin-14 motors such as KlpA and GiKIN14a and will quantitatively explain the available experimental results and provided predicted results, which are critical to the chemo–mechanical coupling mechanism of kinesin-14s.

## 2. Results

For convenience, the tailless KlpA is abbreviated as KlpA-Δtail, KlpA with the insertion of an extra polypeptide linker (3 × GS) in the central region of its neck stalk is abbreviated as KlpA-3 × GS, the tailless GiKIN14a is abbreviated as GiKIN14a-Δtail, and GiKIN14a with the insertion of the extra 3 × GS in the central region of its neck stalk is abbreviated as GiKIN14a-3 × GS, as abbreviated previously [15,16,17]. Throughout, we considered saturating ATP concentrations. When the motor moves on MTs in the minus end direction, it is defined that it moves forward.

### 2.1. Dynamics of the KlpA Motor

#### 2.1.1. The Single KlpA-ΔTail Motor Moving on a Single MT

**Figure 1:**
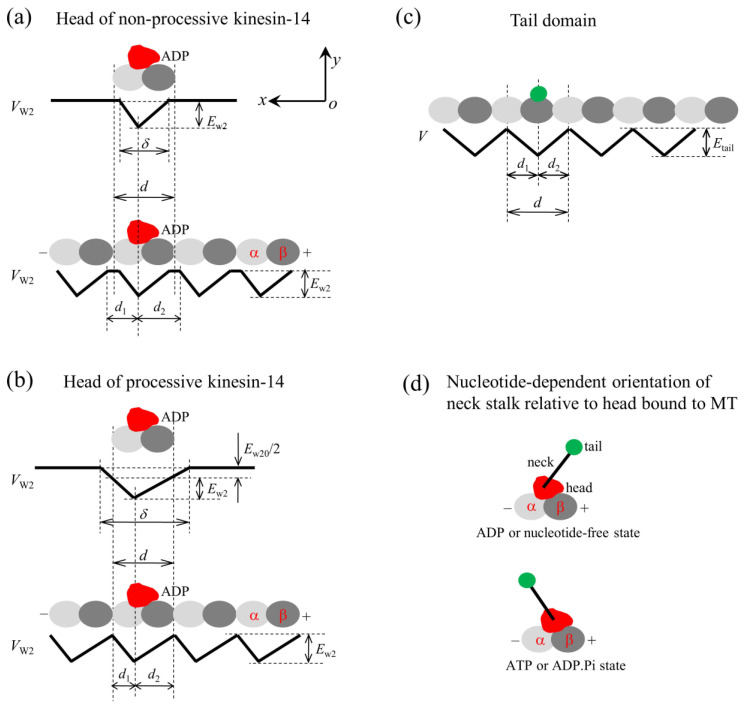
The model for the interaction of the head with MTs and the orientation of the neck stalk for the kinesin-14 motor. The head and tail domain of the motor are drawn in red and green, respectively. (**a**) Interaction potential of the head in the ADP state with a tubulin (**upper** panel) and with an MT filament (**lower** panel) for the non-processive motor. (**b**) Interaction potential of the head in the ADP state with a tubulin (**upper** panel) and with an MT filament (**lower** panel) for the processive motor. (**c**) Interaction potential of the tail domain with an MT filament. (**d**) Two orientations of the neck stalk relative to the head bound to the MT, with the upper panel corresponding to the orientation of the ADP or nucleotide-free state and the lower panel corresponding to the orientation of the ATP or ADP.Pi state.

**Figure 2:**
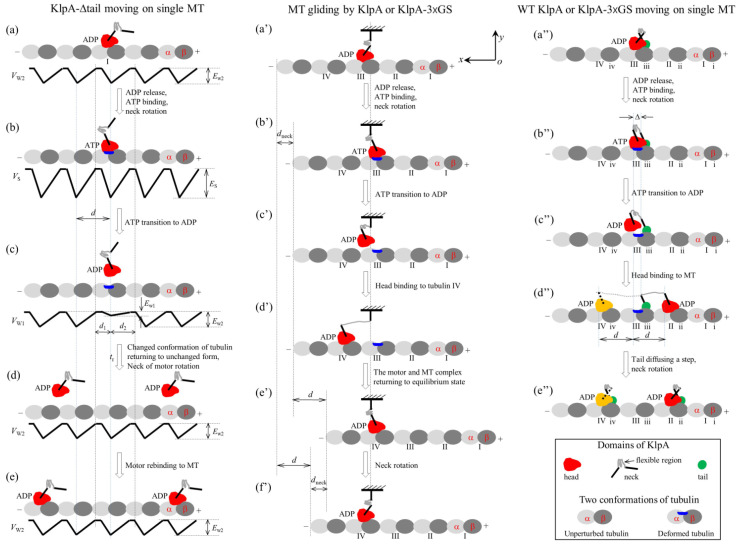
Schematic illustrations of the chemo–mechanical coupling pathway of the KlpA motor. (**a**–**e**) The single KlpA-Δtail motor moving on a single MT (see Section 2.1.1 for detailed descriptions). (**a′**–**f′**) MT gliding by KlpA or the KlpA-3 × GS motor (see Section 2.1.2 for detailed descriptions). (**a″**–**e″**) The single full-length KlpA or KlpA-3 × GS motor moving on a single MT (see Section 2.1.3 for detailed descriptions). In (**d″,e″**), the position of the head drawn in red represents the one where the head has a larger probability to locate and that in yellow represents the one where the head has a smaller probability to locate for the case of full-length KlpA.

For the single KlpA-Δtail motor, only its head can interact with MTs. On the basis of the interaction potential of the head with MTs for the non-processive motor (see Section 4.1 and Figure 1a), the pathway for the single KlpA-Δtail motor moving on a single MT is illustrated schematically in Figure 2a–e.

We start with the motor in its ADP state bound to tubulin I with affinity *E*_w2_ (Figure 2a). After ADP release, followed by ATP binding, but before ATP transition to ADP, the strong interaction between the head and MT causes large conformational changes in the local tubulin I (Figure 2b). After ATP transition to ADP, for a very short time *t*_r_, the ADP head shows a much weaker affinity *E*_w1_ for the local tubulin I than the weak affinity *E*_w2_ for other tubulins with no the conformational changes [19,20]. During time *t*_r_, the head can detach easily from the MT by overcoming the very small affinity *E*_w1_ (Figure 2c). Then, the detached head can diffuse freely far away from the MT. During the time after the motor detaches from the MT and before the motor recontacts the MT, the motor diffuses in a manner with no directional preference (Figure 2d). Upon the motor recontacting the MT surface, the motor binds to the MT with affinity *E*_w2_ (Figure 2e). Then, ADP release, ATP binding and ATP transition to ADP take place, with the motor detaching from the MT and diffusing freely with no directional preference again. Since the tubulin to which the motor rebinds is usually far away along the *x* direction from the tubulin from which the motor detaches, overall, the motor mostly interacts with the MT in a diffusive manner with no apparent directional preference, which is consistent with the prior experimental results [15].

#### 2.1.2. MT Gliding by KlpA or the KlpA-3 × GS Motor

In this section, we consider MT gliding by KlpA or the KlpA-3 × GS motor with its tail domain or the N-terminal end of its neck stalk being surface-immobilized. On the basis of the interaction potential of the head with the MT for the non-processive motor (see Section 4.1 and Figure 1a) and the relative orientation of the neck stalk to the head (see Section 4.2 and Figure 1d), the pathway of the MT gliding by the motor is illustrated schematically in Figure 2a′–f′.

We start with the motor in its ADP state bound to tubulin III on the mobile MT with affinity *E*_w2_ (Figure 2a′). After ADP release and ATP binding, the relatively rigid segment of the neck near the head (this segment being called segment H) rotates to the orientation of the ATP state while the relatively rigid segment near the tail (this segment being called segment T) is kept fixed. The rotation of segment H causes the mobile MT to move in the plus end direction by a distance *d*_neck_ (Figure 2b′).

After ATP transition to ADP, the head detaches from the MT due to the very small affinity *E*_w1_ (Figure 2c′). Considering that the MT is bound by other motors with their neck stalks being connected to the immobilized surface, the detached head can only diffuse relative to the MT in the vicinity of the surface of the MT. For simplicity of analysis but without loss of generality, it is considered here that the MT is kept unmoved during the diffusion of the head relative to the MT. Upon the head diffusing rapidly to the position either at *x* = *d*_1_ and *y* = 0 or at *x* = −*d*_2_ and *y* = 0, the head would fall rapidly into the potential well either at *x* = *d* or at *x* = −*d* due to the large affinity *E*_w2_ that is much larger than *E*_w1_, where we define *x* = 0 and *y* = 0 when the head is on tubulin III (Figure 2b′), and *d*_1_ and *d*_2_ are defined in Figure 1a or Figure 2c. From *x* = 0, the ratio for the head to fall into the potential well at *x* = *d* to that at *x* = −*d* can be determined below.

Suppose that the flexible central region of the neck stalk, which can be stretched elastically, together with other relatively rigid regions of the neck stalk, which can be bent elastically, behave like a linear spring, with the effective elastic coefficient being represented by κ. Firstly, consider the ideal case of κ = 0. The head’s position, *x*, within the range of −*d*_2_ < *x* < *d*_1_, can be expressed as $\langle x^{2}\rangle =2Dt$, with *D* representing motor’s diffusion constant. Hence, the time for the head to reach *x* = *d*_1_ and that to reach *x* = −*d*_2_ can be expressed as $\tau_{10}=d_{1}{\;\;}^{2}/D$ and $\tau_{20}=d_{2}{\;\;}^{2}/D$, respectively. The ratio of the head falling into the potential well at *x* = *d* to that at *x* = −*d* can then be computed with $r_{0}=\tau_{20}/\tau_{10}=d_{2}{\;\;}^{2}/d_{1}{\;\;}^{2}$.

Then, consider the real case of κ > 0. As noted from Figure 2b′–d′, the energy change for the head to move from position *x* = 0 to position *x* = *d* can be expressed as $\Delta \varepsilon_{f}=\kappa d^{2}/2$ while the energy change for the head to move from position *x* = 0 to position *x* = −*d* can also be expressed as $\Delta \varepsilon_{b}=\kappa d^{2}/2$. As performed previously [21,22,23,24,25,26,27,28,29], with these energy changes, the time for the head to fall into the potential well at *x* = *d* and that at *x* = −*d* can be expressed as $t_{10}=\tau_{10}\exp\left(\lambda \beta \Delta \varepsilon_{f}\right)$ and $t_{20}=\tau_{20}\exp\left(\lambda \beta \Delta \varepsilon_{b}\right)$, respectively, where λ ≤ 1 represents the energy-splitting factor and $\beta^{-1}=k_{B}T$ represents the thermal energy, with *k*_B_ being the Boltzmann constant and *T* the absolute temperature. Hence, the ratio of the head falling into the potential well at *x* = *d* (giving a forward step) to that at *x* = −*d* (giving a backward step) can be computed with $r_{1}=t_{20}/t_{10}=r_{0}\exp\left(\lambda \beta \Delta \varepsilon_{b}\right)/\exp\left(\lambda \beta \Delta \varepsilon_{f}\right)$. Substituting the above expressions for *r*_0_, $\Delta \varepsilon_{f}$ and $\Delta \varepsilon_{b}$ into the above expression for *r*_1_, we obtain

(1) $$r_{1}=\alpha^{2},$$

where $\alpha \equiv d_{2}/d_{1}$, characterizing the asymmetry of the interaction potential of the motor with the MT (see Section 4.1), which is called an asymmetric parameter.

From Figure 2c′, if the motor takes a forward step (Figure 2d′), the internal elastic force drives the MT bound by the head to move in the plus end direction by a distance *d* to the position where no internal force is present (Figure 2e′). Then, the neck rotates to the orientation of the ADP state, resulting in the MT moving in the minus end direction by a distance *d*_neck_ (Figure 2f′). Figure 2f′ is the same as Figure 2a′ except that in Figure 2f′, the MT has moved in the plus end direction by a net distance *d* with the hydrolysis of one ATP molecule. Similarly, from Figure 2c′, if the motor takes a backward step the MT moves in the minus end direction by a net distance *d* with the hydrolysis of one ATP molecule.

As noted above, the hydrolysis of one ATP molecule results in the MT moving in either the plus or minus end directions by a distance *d*, with the ratio of the occurrence probability of the plus end movement to that of the minus end movement being equal to *r*_1_. Letting *k* represent the ATPase rate of the motor, the MT gliding velocity can thus be expressed as $v_{1}=kd\left(r_{1}-1\right)/\left(r_{1}+1\right)$. Substituting Equation (1) into the above expression for *v*_1_, we obtain

(2) $$v_{1}=\frac{\alpha^{2}-1}{\alpha^{2}+1}kd.$$

**Figure 3:**
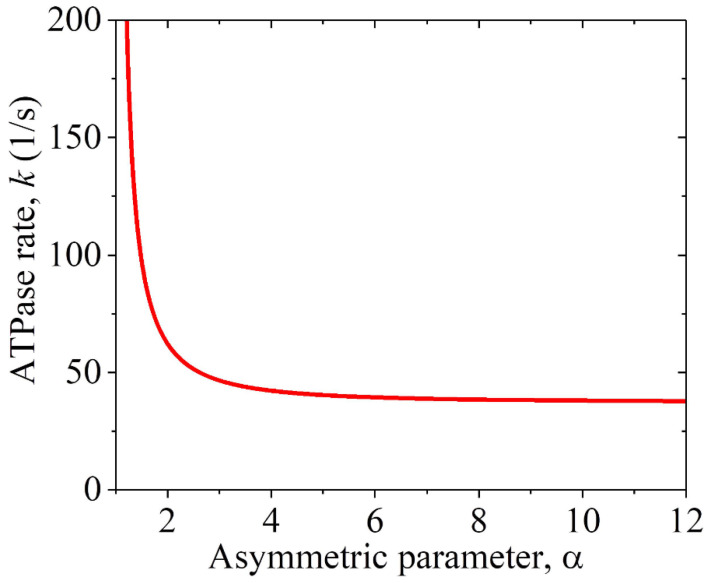
Relationship between the ATPase rate *k* of the KlpA motor and the asymmetric parameter α for the interaction potential of its head with MTs, under which the computed MT gliding velocity *v*_1_ = 298 nm/s.

The prior experimental data showed that the MT gliding velocity by a KlpA motor was *v*_1_ = 309 ± 35 nm/s and that by the KlpA-Δtail motor was *v*_1_ = 287 ± 10 nm/s [15], with the two values being consistent with each other within the experimental errors. This is consistent with our above analysis, showing that the two motors give the same MT gliding velocity. Using Equation (2), we determined the relationship between the ATPase rate *k* and the asymmetric parameter α, under which the computed MT gliding velocity *v*_1_ was equal to the average experimental value of (309 + 287)/2 nm/s = 298 nm/s, as plotted in Figure 3. Note that only under α > 1 can the MT gliding velocity be positive, with the plus end movement of the MT. From Figure 3, it is seen that *k* decreases rapidly with the increase in α and becomes leveled off at a large α. Particularly, *k* decreases only slightly with the increase in α when α > 3. This indicates that to have a high chemo–mechanical coupling efficiency for the motor, the interaction potential of the motor with the MT should have an asymmetric parameter α > 3. Thus, in the following, we take α = 4 (Table 2 and Table 3).

#### 2.1.3. The Single Full-Length KlpA or KlpA-3 × GS Motor Moving on a Single MT

In this section, we consider the single full-length KlpA or KlpA-3 × GS motor moving on a single MT. Due to the flexibility of the central region of the neck stalk, the head and tail can simultaneously bind to a single MT, with the head bound to one filament and the tail bound to the adjacent filament because the neck tilts away from the direction along the filament, namely in the *x* direction [30,31]. On the basis of the interaction potential of the head with the MT for the non-processive motor (see Section 4.1 and Figure 1a) and the relative orientation of the neck stalk to the head (see Section 4.2 and Figure 1d), the pathway for the motor moving on the single MT is illustrated schematically in Figure 2a″–e″.

We start with the head in its ADP state bound to tubulin III with weak affinity *E*_w2_ and the tail bound to binding site iii on the MT (Figure 2a″). Here, it is argued that the N-terminal end of segment H is away from the C-terminal end of segment T by a small distance along the *x* direction (e.g., about 2 nm). After ADP release and ATP binding, segment H rotates to the orientation of the ATP state while segment T is kept fixed (Figure 2b″). Note that during the period of ADP release, ATP binding and the rotation of segment H, the tail can diffuse to either site ii or site iv because of the large diffusion constant of the tail. Since when the tail is at either site ii or site iv, the elastic energy of stretching the neck stalk is much larger than when the tail is at site iii, the tail is nearly always at site iii. Thus, it is a good approximation to consider that at the moment when the rotation of segment H takes place, the tail is at site iii. Considering that the rotation of the relatively rigid neck stalk of the Ncd motor between the orientation of the ADP state and that of the ATP state results in the N-terminal end of the neck stalk moving a distance of about 9~10 nm along the *x* direction [32], it was estimated that the rotation of segment H between the two orientations would result in the N-terminal end of segment H to move a distance of about 4 nm along the *x* direction. Thus, in the state of Figure 2b″, the N-terminal end of segment H would be away from the C-terminal end of segment T by a small distance along the *x* direction, which is represented by Δ (e.g., about 2 nm).

After ATP transition to ADP, the head detaches from the MT by overcoming the very small affinity *E*_w1_ (Figure 2c″). Similar to the above analysis for the position of the tail at the moment when the rotation of segment H takes place, it is also a good approximation to consider that at the moment when ATP transition to ADP takes place, the tail is at site iii. In Figure 2c″, due to the tail binding to the MT, the detached head can only diffuse in the vicinity of the surface of the MT.

As analyzed above for the case of the MT gliding by the motor shown in Figure 2a′–f′, in Figure 2a″–e″ for the ideal case of κ = 0, the time for the head to reach *x* = *d*_1_ and that to reach *x* = −*d*_2_ can be computed with $\tau_{10}=d_{1}{\;\;}^{2}/D$ and $\tau_{20}=d_{2}{\;\;}^{2}/D$, respectively, where *d*_1_ and *d*_2_ are defined in Figure 1a or Figure 2c. The ratio of the head falling into the potential well at *x* = *d* to that at *x* = −*d* can then be computed with $r_{0}=\tau_{20}/\tau_{10}=d_{2}{\;\;}^{2}/d_{1}{\;\;}^{2}$.

Then, consider the real case of κ > 0. As noted from Figure 2b″–d″, the energy change for the head to move from position *x* = 0 to position *x* = *d* can be expressed as $\Delta \varepsilon_{f}=\kappa {\left(d+\Delta \right)}^{2}/2$ while the energy change for the head to move from position *x* = 0 to position *x* = −*d* can be expressed as $\Delta \varepsilon_{b}=\kappa {\left(d-\Delta \right)}^{2}/2$. With these energy changes, the time for the head to fall into the potential well at *x* = *d* and that at *x* = −*d* can be computed with $t_{10}=\tau_{10}\exp\left(\lambda \beta \Delta \varepsilon_{f}\right)$ and $t_{20}=\tau_{20}\exp\left(\lambda \beta \Delta \varepsilon_{b}\right)$, respectively. Hence, the ratio of the head falling into the potential well at *x* = *d* to that at *x* = −*d* can be computed with $r_{2}=t_{20}/t_{10}=r_{0}\exp\left(\lambda \beta \Delta \varepsilon_{b}\right)/\exp\left(\lambda \beta \Delta \varepsilon_{f}\right)$. Substituting the above expressions for *r*_0_, $\Delta \varepsilon_{f}$ and $\Delta \varepsilon_{b}$ into the above expression for *r*_2_, we obtain $r_{2}=\left(d_{2}{\;\;}^{2}/d_{1}{\;\;}^{2}\right)\exp\left(-2\lambda \beta d\kappa \Delta \right)$, which can be re-expressed as

(3) $$r_{2}=\alpha^{2}\exp\left(-2\lambda \beta d\kappa \Delta \right).$$

After the head takes a step (Figure 2d″), due to the large diffusion constant, the tail can diffuse rapidly to either site ii or site iv, where the stretched neck stalk has the minimal elastic energy, and then the neck rotates to the orientation of the ADP state (Figure 2e″). Figure 2e″ is the same as Figure 2a″, except that in Figure 2e″, the motor has taken either a forward or a backward step with the hydrolysis of one ATP molecule.

With the ATPase rate *k* of the head and the stepping ratio *r*_2_, the velocity of the motor moving on the single MT can be computed with $v_{2}=kd\left(r_{2}-1\right)/\left(r_{2}+1\right)$. Substituting Equation (3) into the above expression for *v*_2_, we obtain

(4) $$v_{2}=\frac{\alpha^{2}\exp\left(-2\lambda \beta d\kappa \Delta \right)-1}{\alpha^{2}\exp\left(-2\lambda \beta d\kappa \Delta \right)+1}kd.$$

By comparing Equation (3) with Equation (1), and Equation (4) with Equation (2), it was seen that the stepping ratio and velocity for the MT gliding by KlpA or the KlpA-3 × GS motor corresponded to the stepping ratio and velocity of the single full-length KlpA or KlpA-3 × GS moving on the single MT for the ideal case of κ = 0.

**Figure 4:**
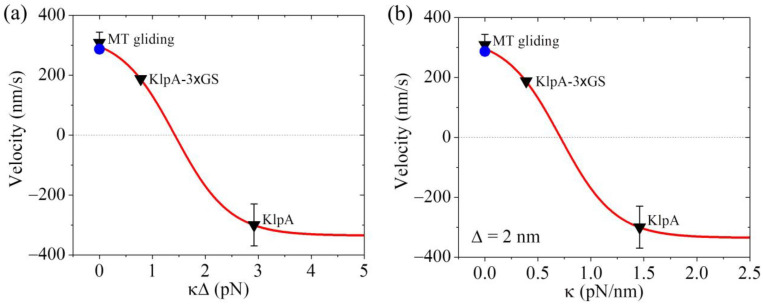
Dynamics of the KlpA motor. Lines represent the theoretical results. Symbols represent experimental data from Popchock et al. [15] and Wang et al. [16], with the black triangles and blue circles for ‘MT gliding’ representing the MT gliding velocity by the KlpA and KlpA-Δtail motors, respectively, and the other two black triangles representing the velocity of the single KlpA-3 × GS and KlpA motors moving on a single MT. Positive velocity represents the plus end movement of the MT in MT gliding or the minus-end-directed movement of the motor on the single MT. (**a**) Velocity of the single KlpA motor moving on a single MT versus κΔ. (**b**) Velocity of the single KlpA motor moving on a single MT versus κ for Δ = 2 nm.

From Equation (4), it is seen that to compute *v*_2_, we need to know the values of parameters λ, α, *k*, Δ and κ. Moreover, it is noted that the product κΔ can be treated as one parameter. As performed previously [21], we took λ = 0.5 throughout. For a given value of α, the value of *k* could be determined from Figure 3 for the KlpA motor. As mentioned above, we took α = 4 (Table 2). In Figure 4a, we show the theoretical results of *v*_2_ versus κΔ, where the positive and negative values of *v*_2_ represent the minus-end-directed and plus-end-directed movements, respectively, and *v*_2_ at κΔ = 0 (or κ = 0) represents the MT gliding velocity, as mentioned just above. For comparison, in Figure 4a, the available experimental data [15,16] for the velocity are also shown. From Figure 4a, it is seen that the theoretical value of *v*_2_ at κΔ = 0.78 pN is consistent with the experimentally measured velocity of the KlpA-3 × GS motor moving on the single MT, and that of *v*_2_ at κΔ = 2.92 pN is consistent with the experimentally measured velocity of the full-length KlpA motor moving on the single MT.

Concretely, we take Δ = 2 nm as an example (Table 2). The theoretical results of *v*_2_ versus κ are shown in Figure 4b, where for comparison, the available experimental data [15,16] are also shown. From Figure 4b, it is seen that KlpA-3 × GS has κ = 0.39 pN/nm while the full-length KlpA has κ = 1.46 pN/nm. This implies that the effective elastic coefficient of the intrinsically flexible central region of the neck stalk together with other relatively rigid regions of the neck stalk for KlpA is about 1.46 pN/nm while the insertion of an extra flexible linker (3 × GS) into the central region of the neck stalk reduces the effective elastic coefficient to a value of about 0.39 pN/nm. This is consistent with our expected results.

Interestingly, from Figure 4b, it is seen that the small κ results in the minus-end-directed movement of KlpA while the large κ results in the plus-end-directed movement. When κ < 0.71 pN/nm, KlpA moves processively toward the minus end and with the decrease in κ, the magnitude of the velocity increases. When κ > 0.71 pN/nm, the motor switches to moving processively toward the plus end and with the increase in κ, the magnitude of the velocity increases. At a high κ, the magnitude of the velocity becomes leveled off. At κ ≈ 0.71 pN/nm, the motor makes unbiased movement. In one word, the elasticity of the neck stalk determines the movement direction of KlpA on a single MT. As the length of the flexible region of the neck stalk sensitively affects κ, it is expected that varying the length of the flexible region will change the velocity and directionality of KlpA. In addition, as the velocity and directionality of KlpA is determined by κ, it is expected that the location of the flexible region in the neck stalk will have little effect on κ and thus have little effect on the velocity and directionality.

Taken together, in this section, we quantitatively explained how the insertion of an extra flexible linker into the central region of the neck stalk can switch the movement direction of the KlpA motor on a single MT, which is due to the decrease in the elasticity of the neck stalk (Figure 4).

#### 2.1.4. KlpA or KlpA-3 × GS Motor Moving Inside Parallel MT Overlap

**Figure 5:**
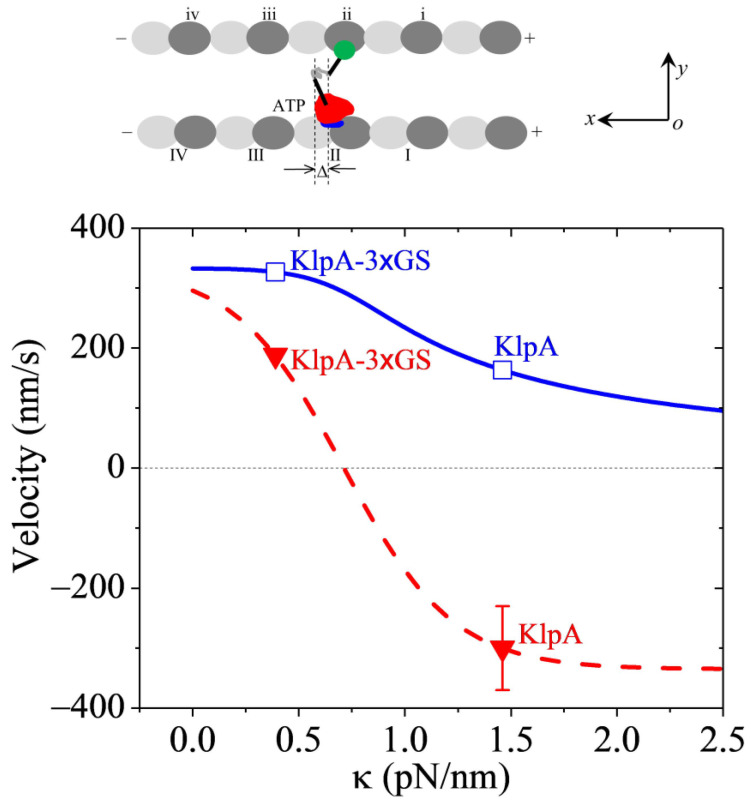
Dynamics of the KlpA motor inside two parallel MTs. (**Upper panel**) illustrates the motor with its head (red) in its ATP state bound to one tubulin on one MT and its tail domain (green) bound to one binding site on the other MT. (**Lower panel**) shows the theoretical results for the velocity of the motor moving inside the MT overlap versus κ (solid blue line), with unfilled squares corresponding to the predicted results for KlpA and KlpA-3 × GS. For comparison, the theoretical results for the velocity of the motor moving on the single MT versus κ are also shown (dashed red line), with filled triangles representing the prior experimental results for KlpA and KlpA-3 × GS [15,16]. A positive velocity represents the motor moving toward the minus end.

In this section, we consider a full-length KlpA or KlpA-3 × GS motor moving inside a parallel MT overlap, with one MT being immobilized and the other MT being mobile (Figure 5, upper panel).

In MT overlap, a lot of motors are present. On average, the force on each MT produced by motors with their heads binding to one MT (called MT-1) and tail domains binding to the other MT (called MT-2) is counteracted by the force produced by motors with their heads binding to MT-2 and tail domains binding to MT-1. Hence, the two parallel MTs cannot move with each other for a large distance but can move with each other for a small distance. Consequently, at the moment when ATP transition to ADP takes place in one motor (called motor-1), the distance Δ (defined in the upper panel of Figure 5) between the N-terminal end of segment H and the C-terminal end of segment T along the *x* direction can be in a range between −4 nm and 4 nm, where Δ is similar to that defined in Figure 2b″. At this moment of ATP transition to ADP taking place, considering that the two MTs are bound by other motors that are still now relative to the MTs, for a given Δ, the movement velocity of motor-1 relative to the two MTs can be computed using Equation (4). For simplicity of analysis, supposing that at the moment of ATP transition to ADP taking place, the values of Δ in a range between $\Delta_{1}$= −4 nm and $\Delta_{2}$= 4 nm are uniformly distributed, the overall velocity of a motor inside a parallel MT overlap can be approximately computed with

(5) $$v_{3}=\frac{\int_{\Delta_{1}}^{\Delta_{2}}\left(\frac{\alpha^{2}\exp\left(-2\lambda \beta d\kappa x\right)-1}{\alpha^{2}\exp\left(-2\lambda \beta d\kappa x\right)+1}\right)dx}{\Delta_{2}-\Delta_{1}}kd.$$

With α = 4 and Δ = 2 nm (see Table 2) and *k* determined from Figure 3, using Equation (5), the computed results of *v*_3_ versus κ are shown in Figure 5 (solid blue line in the lower panel), where for comparison, the computed results (dashed red line) and the prior experimental data (filled red triangles) for the single full-length KlpA and KlpA-3 × GS motors moving on a single MT are reshown. From Figure 5, it is seen that for any value of κ, the motor moves inside the parallel MT overlap toward the minus ends (with *v*_3_ > 0). In particular, the velocity *v*_3_ of KlpA-3 × GS (with κ = 0.39 pN/nm) and that of full-length KlpA (with κ = 1.46 pN/nm) are indicated in Figure 5 by open blue squares. Firstly, it is seen that the full-length KlpA moves inside the parallel MT overlap in the opposite direction to that on a single MT, which is consistent with the prior experimental data [15]. KlpA-3 × GS moves inside the parallel MT overlap in the same direction as that on the single MT. Secondly, it is seen that the velocity of KlpA-3 × GS inside the parallel MT overlap is larger than that on the single MT. The magnitude of the velocity of the full-length KlpA inside the parallel MT overlap is smaller than that on the single MT.

Taken together, in this section, we explained how full-length KlpA can move processively inside parallel MTs toward the minus end whereas it can move on a single MT toward the plus end (Figure 5).

### 2.2. Dynamics of the Single GiKIN14a Motor Moving on the Single MT

#### 2.2.1. The Chemo–Mechanical Coupling Efficiency

**Figure 6:**
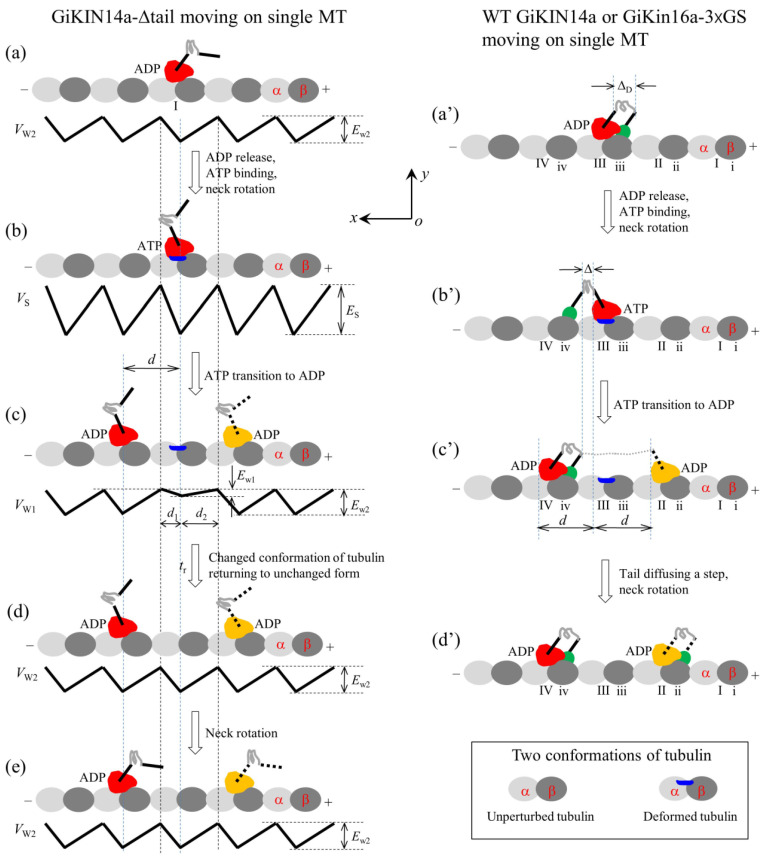
Schematic illustrations of the chemo–mechanical coupling pathway of the GiKIN14a motor. The head of the motor is drawn in red or yellow, while the tail domain is drawn in green. (**a**–**e**) The single GiKIN14a-Δtail motor moving on a single MT (see Section 2.2.1 for detailed descriptions). In (**c**–**e**), the position of the head drawn in red represents the one where the head has a larger probability to locate and that in yellow represents the one where the head has a smaller probability to locate. (**a′**–**d′**) The single full-length GiKIN14a or GiKIN14a-3 × GS motor moving on a single MT (see Section 2.2.1 for detailed descriptions). In (**c′**,**d′**), the position of the head drawn in red represents the one where the head has a larger probability to locate and that in yellow represents the one where the head has a smaller probability to locate.

Firstly, consider the single GiKIN14a-Δtail motor moving on a single MT. For this case, only the head can interact with the MT. On the basis of the interaction potential of the head with the MT for the processive motor (see Section 4.1 and Figure 1b), the pathway for the GiKIN14a-Δtail motor moving on a single MT is illustrated schematically in Figure 6a–e.

We start with the motor in its ADP state bound to tubulin I (Figure 6a). After ADP release and ATP binding but before ATP transition to ADP, the strong interaction between the head and MT causes rapidly large conformational changes in the local tubulin I (Figure 6b). After ATP transition to ADP, within time *t*_r_, the ADP head has a very small affinity *E*_w1_ in the *x* direction and the affinity *E*_w1_ + *E*_w10_/2 + *E*_w20_/2 in the *y* direction for local tubulin I. Thus, the motor has a larger probability to move along the MT filament (the *x* direction) to the neighboring tubulin by overcoming the smaller affinity *E*_w1_ than to detach from the MT by overcoming the larger affinity *E*_w1_ + *E*_w10_/2 + *E*_w20_/2 along the *y* direction (Figure 6c). In time *t*_r_, the local tubulin I elastically returns to its normal unchanged form (Figure 6d). After segment H rotates to the orientation of the ADP state (Figure 6e), a chemo–mechanical coupling cycle is completed. From Figure 6a to e, either a forward or a backward step is made by the hydrolysis of one ATP molecule. Thus, the GiKIN14a-Δtail motor can move processively on the MT, which is consistent with the available experimental data [17].

During the transition from Figure 6b to c, for the ideal case of *E*_w1_ = 0, the time for the head to reach *x* = *d*_1_ and that to reach *x* = −*d*_2_ can be computed with $\tau_{10}=d_{1}{\;\;}^{2}/D$ and $\tau_{20}=d_{2}{\;\;}^{2}/D$, respectively, where *d*_1_ and *d*_2_ are defined in Figure 1b or Figure 6c. For the real case of *E*_w1_ > 0, the time for the head to reach *x* = *d*_1_ and that to reach *x* = −*d*_2_ can be expressed as $t_{10}=\tau_{10}\exp\left(\beta E_{w1}\right)=\exp\left(\beta E_{w1}\right)d_{1}{\;\;}^{2}/D$ and $t_{20}=\tau_{20}\exp\left(\beta E_{w1}\right)=\exp\left(\beta E_{w1}\right)d_{2}{\;\;}^{2}/D$, respectively. As noted, after reaching *x* = *d*_1_ and *x* = −*d*_2_, the head rapidly falls into the potential well of depth *E*_w2_ at *x* = *d* and that at *x* = −*d*, resulting in a forward step and a backward step, respectively. Thus, the forward-to-backward stepping ratio can be computed with $r_{2}=t_{20}/t_{10}=d_{2}{\;\;}^{2}/d_{1}{\;\;}^{2}$, which can be re-expressed as

(6) $$r_{2}=\alpha^{2}.$$

With the stepping ratio *r*_2_, the net number of forward steps per ATP hydrolysis, which is defined as the chemo–mechanical coupling efficiency, can be computed with $E=\left(r_{2}-1\right)/\left(r_{2}+1\right)$. Substituting Equation (6) into the above expression for *E*, we obtain

(7) $$E=\frac{\alpha^{2}-1}{\alpha^{2}+1}.$$

Secondly, consider the single full-length GiKIN14a or GiKIN14a-3 × GS motor moving on a single MT. Due to the flexibility of the central region of the neck stalk, the head and tail can simultaneously bind to the MT, with the head binding to one filament and the tail binding to the adjacent filament. On the basis of the interaction potential of the head with MT for the processive motor (see Section 4.1 and Figure 1b) and the relative orientation of the neck stalk to the head (see Section 4.2 and Figure 1d), the pathway of the motor moving on the single MT is illustrated schematically in Figure 6a′–d′.

We start with the head in its ADP state bound to tubulin III and the tail bound to binding site iii (Figure 6a′), where the neck stalk is minimally stretched. Here, it is argued that the N-terminal end of segment H is away from the C-terminal end of segment T by a small distance along the *x* direction, which is represented by $\Delta_{D}$ (noting that the orientation of segment T for GiKIN14a is distinct from that for KlpA). After ADP release and ATP binding, segment H rotates to the orientation of the ADP state and the tail diffuses to site iv (Figure 6b′), where the neck stalk is minimally stretched. After ATP transition to ADP, the head can diffuse to either tubulin IV or tubulin II (Figure 6c′) (noting that since the energy change, $\Delta E_{\mathrm{neck}}^{\left(T\right)}$, for segment H to rotate from the orientation of the ADP state to that of the ATP state after ATP binding has a very small value approaching zero, the stretching of the neck stalk caused by the forward diffusion of the head can easily induce segment H to rotate to the orientation of the ADP state). If the head has diffused to tubulin II, the tail then diffuses rapidly to site ii, followed by the rotation of segment H (Figure 6d′). From Figure 6a′–d′, either a forward or a backward step is made by hydrolyzing one ATP molecule.

It is noted here that during the diffusion of the head, namely during the transition from Figure 6b′–c′, the binding of the tail to MT can greatly reduce the dissociation rate of the motor from the MT, greatly enhancing the processivity of the motor compared to the case for the GiKIN14a-Δtail motor. Thus, the single full-length GiKIN14a or GiKIN14a-3 × GS motor can move on the MT with a much higher processivity than the GiKIN14a-Δtail motor, which is consistent with the available experimental data [17].

As analyzed above for the GiKIN14a-Δtail motor shown in Figure 6a–e, for the ideal case of κ = 0, the time for the head of the GiKIN14a or GiKIN14a-3 × GS motor in Figure 6a′–d′ to reach *x* = *d*_1_ and that to reach *x* = −*d*_2_ can also be computed with $\tau_{10}=d_{1}{\;\;}^{2}/D$ and $\tau_{20}=d_{2}{\;\;}^{2}/D$, respectively. Then, consider the real case of κ > 0 in Figure 6a′–d′. As noted from Figure 6b′,c′, the energy change for the head to move from *x* = 0 to *x* = *d* can be computed with $\Delta \varepsilon_{f}=\kappa {\left(d/2-\Delta \right)}^{2}/2$, where Δ is the distance between the N-terminal end of segment H and the C-terminal end of segment T along the *x* direction, as indicated in Figure 6b′. Note that in the above expression for $\Delta \varepsilon_{f}$, the rotation of segment H from the orientation of the ATP state to that of the ADP state resulting in the N-terminal end of segment H changing by a distance of about *d*/2 = 4 nm along the *x* direction was considered and for approximation, the energy change $\Delta E_{\mathrm{neck}}^{\left(T\right)}$ for the rotation of segment H was neglected due to $\Delta E_{\mathrm{neck}}^{\left(T\right)}$ approaching zero. The energy change for the head to move from *x* = 0 to *x* = −*d* can be expressed as $\Delta \varepsilon_{b}=\kappa {\left(d+\Delta \right)}^{2}/2$. With these energy changes, the time for the head to fall into the potential well at *x* = *d* and that at *x* = −*d* can be computed with $t_{10}=\tau_{10}\exp\left(\lambda \beta \Delta \varepsilon_{f}\right)$ and $t_{20}=\tau_{20}\exp\left(\lambda \beta \Delta \varepsilon_{b}\right)$, respectively. Thus, the stepping ratio of the head or the stepping ratio of the motor can be computed with $r_{2}=t_{20}/t_{10}=\left(d_{2}{\;\;}^{2}/d_{1}{\;\;}^{2}\right)\exp\left(\lambda \beta \Delta \varepsilon_{b}\right)/\exp\left(\lambda \beta \Delta \varepsilon_{f}\right)$. Substituting the above expressions for $\Delta \varepsilon_{f}$ and $\Delta \varepsilon_{b}$ into the above expression for *r*_2_, we obtain

(8) $$r_{2}=\alpha^{2}\frac{\exp\left[\frac{1}{2}\lambda \beta \kappa {\left(d+\Delta \right)}^{2}\right]}{\exp\left[\frac{1}{2}\lambda \beta \kappa {\left(d/2-\Delta \right)}^{2}\right]}.$$

With the stepping ratio *r*_2_, the net number of the forward steps per ATP hydrolysis, i.e., the chemo–mechanical coupling efficiency of the motor, can be computed with $E=\left(r_{2}-1\right)/\left(r_{2}+1\right)$. Substituting Equation (8) into the above expression for *E*, we obtain

(9) $$E=\frac{\alpha^{2}\exp\left[\frac{1}{2}\lambda \beta \kappa {\left(d+\Delta \right)}^{2}\right]/\exp\left[\frac{1}{2}\lambda \beta \kappa {\left(d/2-\Delta \right)}^{2}\right]-1}{\alpha^{2}\exp\left[\frac{1}{2}\lambda \beta \kappa {\left(d+\Delta \right)}^{2}\right]/\exp\left[\frac{1}{2}\lambda \beta \kappa {\left(d/2-\Delta \right)}^{2}\right]+1}.$$

By comparing Equation (9) with Equation (7), it is seen that the chemo–mechanical coupling efficiency of the GiKIN14a-Δtail corresponds to that of the full-length GiKIN14a or GiKIN14a-3 × GS for the ideal case of κ = 0.

**Figure 7:**
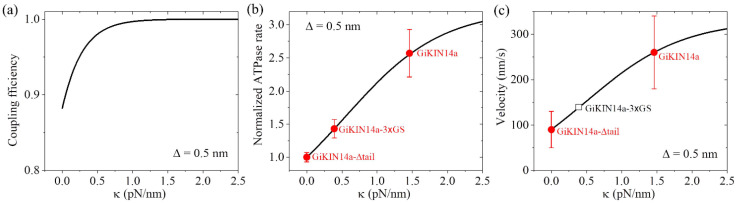
Dynamics of the GiKIN14a motor. Lines represent the theoretical results. (**a**) Chemo–mechanical coupling efficiency versus κ. (**b**) Normalized ATPase rate versus κ. Filled circles represent the experimental data from Tseng et al. [17]. Errors of the experimental data for the full-length GiKIN14a or GiKIN14a-3 × GS motors were computed with $\Delta \bar{k}$ = $\Delta \left(k/k_{0}\right)$ = $\left|\partial \left(k/k_{0}\right)/\partial k\right|\Delta k+\left|\partial \left(k/k_{0}\right)/\partial k_{0}\right|\Delta k_{0}$, where *k* and Δk represent, respectively, the ATPase rate and the corresponding error for the full-length GiKIN14a or GiKIN14a-3 × GS motors, while *k*_0_ and $\Delta k_{0}$ represent, respectively, the ATPase rate and the corresponding error for GiKIN14a-Δtail motor. Error of the experimental data for the GiKIN14a-Δtail motor was computed with $\Delta k_{0}/k_{0}$. (**c**) Velocity versus κ. Filled circles represent the experimental data from Tseng et al. [17]. The unfilled square represents the predicted result. The positive velocity represents the motor moving toward the minus end.

As performed for Figure 4 for KlpA, we also took α = 4 for GiKIN14a (Table 3). We took Δ = 0.5 nm for GiKIN14a (Table 3). The choice of the value of Δ was to make the theoretical results for the ATPase rate of the GiKIN14a and GiKIN14a-3 × GS motors be in agreement with the available experimental results [17] (see next section). Using Equation (9), the computed results of the chemo–mechanical coupling efficiency *E* versus κ are shown in Figure 7a. It is seen that *E* increases with the increase in κ and becomes leveled off to the maximum value of one at a high κ. This implies that the full-length GiKIN14a motor has a larger *E* than the GiKIN14a-3 × GS motor and the latter motor has a larger *E* than the GiKIN14a-Δtail motor. Supposing that the effective elastic coefficient for the neck stalk of GiKIN14a is the same as that of KlpA, from Figure 7a, it is seen that *E* is about 0.88 for the GiKIN14a-Δtail motor at κ = 0, *E* is about 0.97 for the GiKIN14a-3 × GS motor at κ = 0.39 pN/nm (see Figure 4b) and *E* is about 1 for the full-length GiKIN14a motor at κ = 1.46 pN/nm (see Figure 4b).

#### 2.2.2. The ATPase Rate

As noted, when the neck (precisely, segment H) is in the orientation of the ATP state, the nucleotide-binding pocket (NBP) of the head is in its closed form while when segment H is in the orientation of the ADP state, the NBP is in its open form. This is similar to the case for the kinesin-1 head, where in the ATP state, the neck linker is docked and the NBP is in its closed form while in the ADP state, the neck linker is undocked and the NBP is in its open form [33]. The closed NBP activates the ATP transition to ADP, while the open NBP activates ADP release.

In Figure 6b′, with segment H of the ATP head bound to tubulin III in the orientation of the ATP state and the tail at site iv, giving the closed NBP form, the elastic energy of stretching the neck stalk can be expressed as $\kappa \Delta^{2}/2$. If segment H of the ATP head bound to tubulin III rotates to the orientation of the ADP state and the tail is at site iii, giving an open NBP, the elastic energy of stretching of the neck stalk can be expressed as $\kappa \Delta_{D}^{2}/2$. As stated above (see Section 2.2), the energy of segment H of the ATP head being in the orientation of the ATP state and the NBP being in its closed form is $\Delta E_{\mathrm{neck}}^{\left(T\right)}$ larger than that of segment H of the ATP head being in the orientation of the ADP state and the NBP being in its open form. Thus, in Figure 6b′, the probability of the time for segment H in the orientation of the ATP state and the NBP in its closed form can be computed with $\exp\left(-\beta \kappa \Delta^{2}/2-\beta \Delta E_{\mathrm{neck}}^{\left(T\right)}\right)/\left[\exp\left(-\beta \kappa \Delta^{2}/2-\beta \Delta E_{\mathrm{neck}}^{\left(T\right)}\right)+\exp\left(-\beta \kappa \Delta_{D}^{2}/2\right)\right]$. By comparison, in Figure 6b for the GiKIN14a-Δtail motor, the probability of the time for segment H in the orientation of the ATP state and the NBP in the closed form can be expressed as $\exp\left(-\beta \Delta E_{\mathrm{neck}}^{\left(T\right)}\right)/\left[\exp\left(-\beta \Delta E_{\mathrm{neck}}^{\left(T\right)}\right)+1\right]$. Therefore, the rate of ATP transition to ADP in the pathway of Figure 6a′–d′ for the full-length GiKIN14a or GiKIN14a-3 × GS motors normalized by that in the pathway of Figure 6a–e for the GiKIN14a-Δtail motor can be expressed as

(10) $$\bar{k}=\frac{\exp\left(-\frac{1}{2}\beta \kappa \Delta^{2}-\beta \Delta E_{\mathrm{neck}}^{\left(T\right)}\right)/\left[\exp\left(-\frac{1}{2}\beta \kappa \Delta^{2}-\beta \Delta E_{\mathrm{neck}}^{\left(T\right)}\right)+\exp\left(-\frac{1}{2}\beta \kappa \Delta_{D}^{2}\right)\right]}{\exp\left(-\beta \Delta E_{\mathrm{neck}}^{\left(T\right)}\right)/\left[\exp\left(-\beta \Delta E_{\mathrm{neck}}^{\left(T\right)}\right)+1\right]},$$

where κ = 0, giving $\bar{k}$ = 1, which corresponds to the case for the GiKIN14a-Δtail motor with the pathway of Figure 6a–e. Since ADP release is the non-rate-limiting step of ATPase activity, the ATPase of the motor in the pathway of Figure 6a′–d′ normalized by that in the pathway of Figure 6a–e can also be computed using Equation (10).

As in Figure 7a, we took Δ = 0.5 nm (Table 3). Considering that the rotation of segment H between the orientation of the ADP state and that of the ATP state results in the N-terminal end of segment H moving a distance of about 4 nm along the *x* direction, as mentioned in Section 2.1.3, we took $\Delta_{D}$ = 3.5 nm (Table 3). We took $\Delta E_{\mathrm{neck}}^{\left(T\right)}$ = 0.8*k_B_T* (Table 3), which was very small (noting that the small positive value of $\Delta E_{\mathrm{neck}}^{\left(T\right)}$ indicates that even in an ATP state, the neck stalk has a slightly larger probability in the orientation of the ADP state). The choice of the value of $\Delta E_{\mathrm{neck}}^{\left(T\right)}$ was to make the theoretical results for the ATPase rate of the GiKIN14a and GiKIN14a-3 × GS motors be in agreement with the available experimental results [17]. Using Equation (10), the computed results of the normalized ATPase rate versus κ are shown in Figure 7b. For comparison, in Figure 7b, we also show the available experimental data [17], where the GiKIN14a-Δtail motor has κ = 0, GiKIN14a-3 × GS motor has κ = 0.39 pN/nm while the full-length GiKIN14a has κ = 1.46 pN/nm, as mentioned above for the results of Figure 7a. From Figure 7b, it is seen that the theoretical results are in good agreement with the available experimental data [17].

#### 2.2.3. The Velocity

With the chemo–mechanical coupling efficiency *E*, which is given by Equation (9), and the normalized ATPase rate $\bar{k}$, which is given by Equation (10), the velocity of the single GiKIN14a motor moving on a single MT can be computed with $v_{2}=E\bar{k}k_{0}d$, where *k*_0_ is the ATPase rate of the GiKIN14a-Δtail motor. Substituting Equations (9) and (10) into above expression for *v*_2_, we obtain

(11) $$\begin{array}{ll} v_{2} & =\frac{\alpha^{2}\exp\left[\frac{1}{2}\lambda \beta \kappa {\left(d+\Delta \right)}^{2}\right]/\exp\left[\frac{1}{2}\lambda \beta \kappa {\left(d/2-\Delta \right)}^{2}\right]-1}{\alpha^{2}\exp\left[\frac{1}{2}\lambda \beta \kappa {\left(d+\Delta \right)}^{2}\right]/\exp\left[\frac{1}{2}\lambda \beta \kappa {\left(d/2-\Delta \right)}^{2}\right]+1}\\ & \times \frac{\exp\left(-\frac{1}{2}\beta \kappa \Delta^{2}-\beta \Delta E_{\mathrm{neck}}^{\left(T\right)}\right)/\left[\exp\left(-\frac{1}{2}\beta \kappa \Delta^{2}-\beta \Delta E_{\mathrm{neck}}^{\left(T\right)}\right)+\exp\left(-\frac{1}{2}\beta \kappa \Delta_{D}^{2}\right)\right]}{\exp\left(-\beta \Delta E_{\mathrm{neck}}^{\left(T\right)}\right)/\left[\exp\left(-\beta \Delta E_{\mathrm{neck}}^{\left(T\right)}\right)+1\right]}k_{0}d \end{array}.$$

With parameter values α = 4 and Δ = 0.5 nm (Table 3), as in Figure 7a, and parameter values $\Delta E_{\mathrm{neck}}^{\left(T\right)}$ = 0.8*k_B_T* and $\Delta_{D}$ = 3.5 nm (Table 3), as in Figure 7b, using Equation (11), the computed results of the velocity *v*_2_ versus κ are shown in Figure 7c, where *k*_0_ = 12.8 $s^{-1}$ for the ATPase rate of the GiKIN14a-Δtail motor, which can be determined similarly to that for KlpA using Figure 3. Note that interestingly, this value of *k*_0_ = 12.8 $s^{-1}$ is close to the available experimental datum of 10.0 ± 0.7 $s^{-1}$ [17]. Since the experimental value of 10.0 ± 0.7 $s^{-1}$ was measured from a bulky assay whereas the theoretical value of *k*_0_ = 12.8 $s^{-1}$ was obtained from a fit to single-molecule data, it is reasonable that the former result is slightly smaller than the latter. From Figure 7c, it is also interesting that the theoretical results are in good agreement with the available experimental data [17], where the GiKIN14a-Δtail motor has κ = 0 and the full-length GiKIN14a has κ = 1.46 pN/nm, as mentioned above in Figure 7a,b. For the GiKIN14a-3 × GS motor at κ = 0.39 pN/nm, the predicted velocity was about 140 nm/s, which could be tested easily in future experiments.

Taken together, in this section, we quantitatively explained how the tail domain and neck stalk can accelerate the ATPase rate and velocity of the GiKIN14a motor during its processive movement on a single MT. With only two adjustable parameters Δ and $\Delta E_{\mathrm{neck}}^{\left(T\right)}$ (see Table 3), the theoretical results are in good agreement with the available experimental data (Figure 7b,c) [17].

## 3. Discussion

### 3.1. Origin of Full-Length Ncd Being Incapable of Diffusing with a Directional Preference Inside Parallel MT Overlaps Contrary to Ncd-3 × GS Being Capable of Diffusing with a Directional Preference toward the Minus Ends

In the experiments of Wang et al. [16], the dynamics of the full-length Ncd and Ncd-3 × GS motors in parallel MT overlaps were also studied, where the two parallel MTs were firstly cross-linked by full-length KlpA motors, and then, either full-length Ncd or the Ncd-3 × GS motors were introduced. Intriguingly, it was found that the full-length Ncd can preferentially accumulate in the MT overlap region over time, showing no preferential accumulation at either the minus or plus ends, and in contrast, the Ncd-3 × GS cannot show preferential accumulation in the overlap region and instead can strongly accumulate at the minus ends. Based on the studies in this paper, these intriguing experimental results can be explained as follows.

First, consider the full-length Ncd with a relatively rigid neck stalk. As the equilibrium position of the tail domain relative to the head along the MTs for Ncd can be different from that for KlpA, when the tail of one Ncd is bound to one MT (called MT-1) the detached head is usually deviated away by a small distance from its binding site on the other MT (called MT-2) and the orientation of the detached head is deviated away by an angle from that of the head bound to MT-2. Thus, in order for the detached head to bind to the binding site on MT-2, the relatively rigid neck of the Ncd is required to bend largely. Due to the rigidity of the neck, the head will have a slow rate to bind to MT-2. During the long time period after the head detaches from MT-2 and before it rebinds to MT-2, the tail will carry out unbiased diffusion on MT-1 over a long distance. Since the tubulin to which the head rebinds is usually far away from the tubulin from which the head detaches, the full-length Ncd motor will overall show no directionally preferential movement inside the MT overlap. Since the tail has a much slower rate to move out of the MT end than that to move onto the MT lattice [21], the full-length Ncd motor is preferentially confined inside the overlap. These are consistent with the prior experimental results [16].

Second, consider the Ncd-3 × GS with a flexible central region in its neck stalk. After the head detaches from one tubulin on MT-2, the head can bind rapidly (in an order of microseconds) to the neighboring tubulin on MT-2 by easily stretching its neck stalk. Thus, as in the case of KlpA-3 × GS, as studied in Figure 5, Ncd-3 × GS will move preferentially toward the minus ends of parallel MTs. After reaching the minus ends, since the tail has a very small rate to move out of the end, the Ncd-3 × GS will accumulate at the minus ends. These are also consistent with the prior experimental results [16].

Moreover, it is noted that Ncd-3 × GS will show similar dynamical behavior to KlpA-3 × GS in MT gliding and in its motility on a single MT. Therefore, the dynamics of Ncd-3 × GS will be similar to that of KlpA-3 × GS, as presented in Figure 3, Figure 4 and Figure 5.

### 3.2. Difference between the Origin of the Bidirectional Movement of Kinesin-14 and That of Kinesin-5

As prior experimental studies have shown, the single kinesin-14 KlpA containing a tail domain shows bidirectional movement on a single MT, which is modulated by the central region of its neck stalk [15,16]. The WT motor moves processively toward the MT plus end whereas the motor, with an insertion of an extra flexible linker (3 × GS) into the central region, moves processively toward the minus end. Here, the bidirectional movement of KlpA is explained theoretically, which is determined by two parameters—the effective elastic coefficient κ for the neck stalk and the asymmetric parameter α for the interaction potential of the head with the MT. For a small κ, the movement direction is mainly determined by α, with α > 1 giving the minus-end-directed movement. For a large κ, the movement direction is mainly determined by the change in the elastic energy of the stretching of the stalk for the head to take a plus-end-directed step relative to that to take a minus-end-directed step. For KlpA-3 × GS, κ is small and thus the motor moves toward the minus end due to α > 1. For WT KlpA, the change in the elastic energy of the stretching of the stalk for the head to take a plus-end-directed step is evidently smaller than that to take a minus-end-directed step, making the plus-end-directed stepping rate larger than the minus-end-directed stepping rate. Thus, the motor moves overall toward the plus end.

By comparison, the prior experimental data showed that some yeast kinesin-5 motors such as *S. cerevisiae* Cin8 and Kip1 and *S. pombe* Cut7 also showed bidirectional movement on a single MT, which was modulated by the ionic strength in the solution [34,35,36,37,38]. Under high or physiological ionic strength, the single kinesin-5 motor moves processively toward the minus end, whereas under low ionic strength, it moves processively toward the plus end. The bidirectional movement of kinesin-5 was explained theoretically before [39,40], which can be redescribed briefly as follows.

It was proposed that the front or plus end head with its neck linker in the minus end direction has a larger ATPase rate than the rear head with its neck linker in the plus end direction, and the front head has a larger *E*_w1_ for its local tubulin than the rear head for its local tubulin. First, consider the high ionic strength. Under this condition, both the front and rear heads have very small values of *E*_w1_. Thus, after ATP transition to ADP in one head, the head can detach with a nearly 100% probability from its local tubulin by overcoming the very small affinity *E*_w1_, diffuse past the MT-bound head and bind to the nearest tubulin with affinity *E*_w2_. Therefore, after ATP transition to ADP occurs in the front head, the dimeric motor makes a minus-end-directed step, while after ATP transition to ADP occurs in the rear head, the motor makes a plus-end-directed step. Since the front head has a larger ATPase rate than the rear head, the motor overall moves toward the minus end. Second, consider the low ionic strength. Under this condition, the values of *E*_w1_ become larger than those under the high ionic strength. Thus, after ATP transition to ADP occurs in the front head, the head can have a very small probability to detach from its local tubulin due to the relatively large value of *E*_w1_, resulting in a futile chemo–mechanical coupling cycle occurring with a very large probability and accordingly a minus-end-directed step occurring with a very small probability. By contrast, after ATP transition to ADP occurs in the rear head, the head can still have a large probability to detach from its local tubulin because the rear head has a smaller *E*_w1_ than the front head, resulting in a plus-end-directed step occurring with a large probability. Therefore, the dimeric motor overall can move toward the plus end.

## 4. The Model

For a homodimeric kinesin-14 motor, because the flexible neck linker joining the head and coiled coil neck stalk is quite short and the C-terminus of the neck stalk, to which the two neck linkers are joined, is too stable to disrupt under a rupture force that is not too large [41,42], the two heads of the motor are unable to interact simultaneously with the same MT. Hence, at any one time, only one of the two heads is able to interact with a single MT. For simplicity, in all graphics shown in this paper, only one head is drawn. Similar to that proposed before [21,43], the model for the motor is stated briefly below.

### 4.1. Interaction Potentials of the Motor with MTs

For a non-processive kinesin-14 motor such as KlpA, with a tailless construct capable of moving non-processively on a single MT, the interaction potential of the head with an isolated tubulin is shown in the upper panel of Figure 1a, with the affinity of the head in its ADP state for tubulin being *E*_w2_ and the interaction distance of the head with tubulin in the *x* direction, δ, being shorter than the MT filament period *d* (=8 nm). From this potential, it was deduced that the interaction potential of the head with an MT filament has the form shown in the lower panel of Figure 1a, with the affinity of the head for tubulin in the filament being *E*_w2_ in both the *x* and *y* directions. The ratio $\alpha \equiv d_{2}/d_{1}$ characterizes the asymmetry of this potential, with α = 1 corresponding to the symmetrical potential.

For a processive kinesin-14 motor such as GiKIN14a, with a tailless construct capable of moving processively on a single MT, the interaction potential of the head with an isolated tubulin is shown in the upper panel of Figure 1b, with the affinity of the head in the ADP state for the tubulin being *E*_w2_ + *E*_w20_/2 and the interaction distance of the head with the tubulin in the *x* direction, δ, being longer than *d*. From this potential, it was deduced that the interaction potential of the head with an MT filament has the form shown in the lower panel of Figure 1b, with the affinity of the head for tubulin in the filament being *E*_w2_ and *E*_w2_ + *E*_w20_ in the *x* and *y* directions, respectively, where the affinity in the *y* direction outside the region of tubulin along the filament should be the sum of the extra affinity *E*_w20_/2 to one tubulin outside the region of the tubulin and that to the adjacent tubulin. The ratio $\alpha \equiv d_{2}/d_{1}$ characterizes the potential asymmetry.

The Interaction strength of the head with MTs is dependent on the nucleotide state of the head. In the ADP state, the interaction is weak, while in other nucleotide states, the interaction is strong [44,45]. The strong interaction can cause large conformational changes in local tubulin [19,20,46,47,48,49,50,51], while the weak interaction has little effect on the tubulin conformation [19,20]. The ADP head shows a much lower affinity for tubulin of large conformational changes than tubulin of no or little conformational changes [19,20]. For instance, for the non-processive kinesin-14 motor, in a cycle of ATPase activity, the temporal evolution of the affinity between the head and MTs is stated below (see, e.g., Figure 2a–e). In the empty and ATP states, the affinity (*E*_S_) is strong. After the ATP transition to ADP, with the head transiting to the conformation of the ADP state, for a very short time *t*_r_, local tubulin can still retain the large conformational changes caused by the interaction with the head in the strong MT-binding state. Hence, within time *t*_r_, the ADP head shows a much weaker affinity (*E*_w1_) for the local tubulin than its weak affinity (*E*_w2_) for other tubulins with no or little conformational changes [19,20]. In time *t*_r_, the local tubulin returns elastically to its normal unchanged form, with the affinity of the local tubulin for the ADP head changing to *E*_w2_.

The interaction between the tail domain and MTs is independent of the nucleotide state of the head, with the interaction potential being shown in Figure 1c. The interaction distance of the tail with an isolated binding site on MTs in the *x* direction, δ, is longer than *d*, giving the affinity of the tail for a binding site in a MT filament being *E*_tail_ and *E*_tail_ + *E*_tail0_ in the *x* and *y* directions, respectively, where the period of the interaction potential of the tail with the MT filament being equal to the period (*d*) of tubulins on the filament (Figure 1c). From this potential (Figure 1c), it is expected that the truncated kinesin-14, having only a tail domain, can diffuse on the MT filament with a large diffusion constant due to the smaller affinity *E*_tail_ in the *x* direction but with a small rate to dissociate due to the larger affinity *E*_tail_ + *E*_tail0_ in the *y* direction, as the available experimental data showed for the truncated HSET [52] and GiKIN14a [17].

### 4.2. Orientations of the Neck Stalk Relative to the Head and Tail Domain

The available structural data for Ncd and Vik1-Kar3 showed that when the head is in the ADP or empty state, the orientation of the neck stalk relative to the head bound to MTs is schematically represented in the upper panel of Figure 1d, while when the head is in the ATP state, the orientation of the neck stalk relative to the head bound to MTs is schematically represented in the lower panel of Figure 1d [30,31]. Throughout, we used ATP to represent both ATP and ADP.Pi. According to these structural data, it was deduced that for a kinesin-14 motor, two orientations of the neck stalk relative to the head are present. One is defined as the orientation of the ADP or empty state (upper panel of Figure 1d), and the other one is defined as the orientation of the ATP state (lower panel of Figure 1d). Note that the available structural data for Ncd and Vik1-Kar3 showed that the neck in any nucleotide state tilts away in the same direction from the *x* direction [30,31], which is not shown here.

Let $\Delta E_{\mathrm{neck}}^{\left(D\right)}$ represent the energy change for the neck to rotate from the orientation of the ATP state to that of the ADP state when the head is bound with ADP, and let $\Delta E_{\mathrm{neck}}^{\left(T\right)}$ represent the energy change for the neck to rotate from the orientation of the ADP state to that of the ATP state when the head is bound with ATP. For KlpA, it is argued here that $\Delta E_{\mathrm{neck}}^{\left(D\right)}$ and $\Delta E_{\mathrm{neck}}^{\left(T\right)}$ have large negative values. This means that after the neck of KlpA rotates to the orientation of the ATP (ADP) state, which is induced by ATP binding (ATP transition to ADP), the neck stalk can be kept stably in the orientation of the ATP (ADP) state under a force that is not too large on the neck before ATP transition to ADP (ATP binding). For GiKIN14a, it is argued here that $\Delta E_{\mathrm{neck}}^{\left(T\right)}$ has a very small value approaching zero. This means that after ATP binding, the neck of GiKIN14a can transit rapidly between the orientation of the ADP state and that of the ATP state.

For the kinesin-14 motor containing an intrinsically flexible central region in the neck stalk, such as KlpA and GiKIN14a, it is argued here that the orientation of the neck stalk relative to the tail domain is kept fixed, independent of the nucleotide state of the head. KlpA and GiKIN14a have distinct orientations of the neck stalk relative to the tail domain.

## 5. Conclusions

In summary, we theoretically studied the dynamics of kinesin-14 motors, such as KlpA, KlpA-3× GS, GiKIN14a, GiKIN14a-3× GS, etc., having either an intrinsically flexible neck stalk or a flexible neck stalk caused by the insertion of an extra polypeptide linker in the central region. The theoretical results quantitatively explain the available experimental results. We explained the mechanism of single full-length KlpA being capable of moving processively on a single MT toward the plus end whereas single KlpA-3× GS is capable of moving processively on a single MT toward the minus end. The mechanism behind the full-length KlpA being able to move processively inside parallel MTs toward the minus ends was also explained. The mechanism of the tail domain being capable of accelerating the ATPase rate and velocity of the GiKIN14a motor during its processive movement on a single MT was furthermore explained. Additionally, the origin of the full-length Ncd being incapable of diffusing with a directional preference contrary to the Ncd-3× GS being capable of diffusing with a directional preference toward the minus ends of parallel MTs was discussed. Finally, the difference between the origin of the bidirectional movement of the kinesin-14 KlpA motor and that of some kinesin-5 motors on a single MT was also discussed.

## Figures and Tables

**Table 1:** Overview of published experimental results on the dynamics of KlpA.

Form of KlpA	Dynamical Feature	Reference
Tailless KlpA (KlpA-Δtail)	Unbiased diffusion on a single MT	Ref. [15]
Full-length KlpA (KlpA)	Minus-end-directed motion in MT gliding	Ref. [15]
Full-length KlpA (KlpA)	Minus-end-directed motion inside parallel MTs	Ref. [15]
Full-length KlpA (KlpA)	Plus-end-directed motion on a single MT	Ref. [15]
KlpA with the insertion of 3 × GS (KlpA-3 × GS)	Minus-end-directed motion on a single MT	Ref. [16]

**Table 2:** Parameter values for KlpA.

Parameter	Value	Description
α	4	Determined theoretically
Δ	2 nm	Adjustable

**Table 3:** Parameter values for GiKIN14a.

Parameter	Value	Description
α	4	Determined theoretically
Δ	0.5 nm	Adjustable
$\Delta_{D}$	3.5 nm	Determined from Δ
$\Delta E_{\mathrm{neck}}^{\left(T\right)}$	0.8 *k_B_T*	Adjustable

## Data Availability

Data are contained within the article.
